# Plasma Device Functions and Tissue Effects in the Female Pelvis—A Systematic Review

**DOI:** 10.3390/cancers15082386

**Published:** 2023-04-20

**Authors:** Nick J. van de Berg, Gatske M. Nieuwenhuyzen-de Boer, Xu Shan Gao, L. Lucia Rijstenberg, Heleen J. van Beekhuizen

**Affiliations:** 1 Department of Gynaecological Oncology, Erasmus MC Cancer Institute, 3015 GD Rotterdam, The Netherlands; 2 Department of Biomechanical Engineering, Delft University of Technology, 2628 CD Delft, The Netherlands; 3 Department of Obstetrics and Gynaecology, Albert Schweitzer Hospital, 3318 AT Dordrecht, The Netherlands; 4 Division of Reproductive Endocrinology and Infertility, Department of Obstetrics and Gynaecology, Erasmus University Medical Center, 3015 GD Rotterdam, The Netherlands; 5 Department of Pathology, Erasmus University Medical Center, 3015 GD Rotterdam, The Netherlands

**Keywords:** argon plasma coagulation, helium plasma coagulation, argon beam coagulator, cold atmospheric plasma, neutral argon plasma, thermal spread, thermal effects depth, vaporization depth

## Abstract

**Simple Summary:**

Plasmas are used in various forms and with various functions in gynaecology. Knowledge on plasma–tissue effects and target coverage is crucial to achieve complete treatment, reduce recurrence, and limit damage to healthy tissues. In this review, historical and future plasma applications are summarized, and the depths of (non-)thermal effects are evaluated in tissues of the female pelvis. A literature search was performed in the Medline Ovid, Embase, Cochrane, Web of Science, and Google Scholar databases. Fourteen articles were found with data on thermal effects depth. For plasma-assisted electrosurgical devices, depths (<2.4 mm) relied on current dispersion in tissue. In turn, for electrically neutral argon plasma, depths remained superficial (<1.0 mm). The depth and uniformity of cold atmospheric plasma effects requires further investigation. This review identifies upcoming and potentially high-gain applications of plasma in the field of gynaecology, of which the therapeutic effectiveness must be examined in translational and clinical studies.

**Abstract:**

Medical use of (non-)thermal plasmas is an emerging field in gynaecology. However, data on plasma energy dispersion remain limited. This systematic review presents an overview of plasma devices, fields of effective application, and impact of use factors and device settings on tissues in the female pelvis, including the uterus, ovaries, cervix, vagina, vulva, colon, omentum, mesenterium, and peritoneum. A search of the literature was performed on 4 January 2023 in the Medline Ovid, Embase, Cochrane, Web of Science, and Google Scholar databases. Devices were classified as plasma-assisted electrosurgery (ES) using electrothermal energy, neutral argon plasma (NAP) using kinetic particle energy, or cold atmospheric plasma (CAP) using non-thermal biochemical reactions. In total, 8958 articles were identified, of which 310 were scanned, and 14 were included due to containing quantitative data on depths or volumes of tissues reached. Plasma-assisted ES devices produce a thermal effects depth of <2.4 mm. In turn, NAP effects remained superficial, <1.0 mm. So far, the depth and uniformity of CAP effects are insufficiently understood. These data are crucial to achieve complete treatment, reduce recurrence, and limit damage to healthy tissues (e.g., prevent perforations or preserve parenchyma). Upcoming and potentially high-gain applications are discussed, and deficits in current evidence are identified.

## 1. Introduction

Plasma is an energetic state of matter consisting of ions and free electrons, typically created by electric discharges in a plasma medium. In the early 1990s, plasma-based devices were first used in the management of ovarian cancers, because of the improved control of thermal depth of injury compared to monopolar electrosurgery (ES) [1]. This controllability makes the devices suitable for complete cytoreductive surgery and for producing haemostasis on the colon, mesenterium, ureters and diaphragm [1,2]. Over the years, many other applications of plasma-based devices have been proposed, evaluated, and used in gynaecology.

Current plasma-based devices are summarized in Table 1 and include the Argon Beam Coagulator (ABC), Argon Plasma Coagulation (APC), J-Plasma, Helica Thermal Coagulator (HTC), Martin Argon-Beamer System (MABS), PlasmaBlade, PlasmaJet (PJ), kINPen, and PlasmaDerm. Most of these devices (ABC, APC, J-Plasma, HTC, MABS, PlasmaBlade) rely on ES. Typically, they do not use plasma-tissue effects directly, but the plasma does help achieve a more even dispersion of electrical energy in tissue. The PJ uses kinetic plasma particle energy to thermally treat tissue. To emphasize the difference with the former group, this device is sometimes referred to as electrically neutral argon plasma (NAP). The kINPen and PlasmaDerm use cold atmospheric plasmas (CAP) and rely on biochemical plasma-tissue effects. Cold plasmas can be administered to tissue through atmospheric pressure plasma jets (APPJ), beams, torches, or plumes; via dielectric barrier discharges (DBD); or via plasma-activated media (PAM), e.g., gases or liquids (PAL).

Definitions for the terms hot and cold in plasma medicine deviate from those in basic plasma sciences. Hot refers to the ability to cause thermal damage to tissue. This can include coagulation, desiccation, ablation, or charring. Irreversible thermal damage in tissue starts at temperatures as low as 43 °C and is a function of temperature and exposure time [3,4]. In turn, cold plasma effects are dominated by non-thermal biochemical responses with reactive oxygen and nitrogen species. The terminology becomes somewhat confusing in plasma-assisted ES, for which cold plasmas may be used to control electrothermal (ohmic) heating of tissue [5].

The aim of this review of the literature is to compare medical applications and tissue effects of plasma devices used in the female pelvis and in gynaecology. This is needed to stay up-to-date with evidence-based and upcoming practices in plasma medicine, to stimulate research to address deficits in current evidence, and to guide technologies towards their most effective clinical use. This review focuses on plasma-applications on tissues of the uterus, ovaries, cervix, vagina, vulva, colon, omentum, mesenterium, and peritoneum.

## 2. Materials and Methods

**Figure 1:**
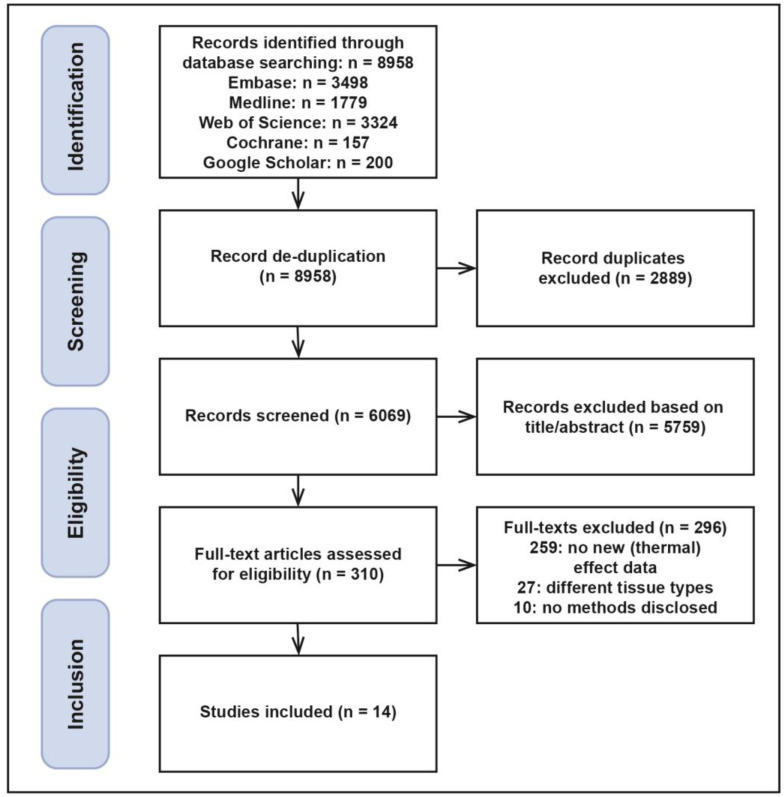
PRISMA flowchart indicating the selection of articles for this systematic review.

A search for literature on quantitative (thermal) effects of plasma-based devices in gynaecological and pelvic tissues was performed in Medline Ovid, Embase, Cochrane, Web of Science, and Google Scholar. This review was not registered on PROSPERO. Search methods and results are illustrated in the PRISMA diagram in Figure 1. Full-text manuscripts published before 4 January 2023 were included. The search was undertaken using key terms, including: “argon plasma coagulation”, “plasmajet”, “cytoreductive surgery” and “tissue damage”. Article titles and texts were scanned independently by X.S.G. and N.J.v.d.B., followed by a discussion to reach consensus on inclusions. Article titles, abstracts, and full texts were reviewed to extract data on the extent of tissue effects. For all studies, the following data were obtained: tissue type, number of lesions, power, exposure time, device–tissue distance, and quantitative values of tissue effects reported.

## 3. Results

In total, 8958 articles were identified. After removing duplicates, an initial pool of 6069 articles remained, of which the titles and abstracts were scanned. Of the remaining 310 records, full texts were retrieved and checked for the description of (thermal) effect data on the tissue types included. In total, 14 articles were identified with new quantitative data on energy dispersion of plasma devices.

### 3.1. Depth of Tissue Effects

An overview of the depth of tissue effects of plasma-based devices is shown in Table 2. These include thermal effects for plasma-assisted ES and NAP devices. Thermal effects are typically measured in formalin-fixed and haematoxylin- and eosin-stained specimens. Metrics included the total depth of damage (**TDD**), or its constituents: the “depth of the crater”, ablation depth or vaporization depth (**VD**), and the thermal effects depth (**TED**). The latter group may again be divided in layers of carbonized eschar and coagulative necrosis [6]. Few articles also measured the width or lateral spread of thermal effects. The spread of non-thermal effects of CAP effects is discussed briefly.

#### 3.1.1. Electrosurgical Devices

Electrosurgical devices (ABC, APC, HTC, J-plasma) use beams of ionized argon or helium for a diffuse conduction of electricity to tissue. The prime tissue effects depend not on interactions with plasma but on ohmic heating. For the ABC, TDD was related to power setting (60–100 W) and tissue interaction time (1–5 s) and ranged between 1.7–5.6 mm in a series of 144 epithelial ovarian carcinoma specimens collected from a single patient [6]. Power and time had a high impact on VD and a lower impact on TED. Notably, the depth ratios of eschar and necrosis layers were highly consistent. The depth and width of necrosis caused by the ABC device were also assessed in porcine uterine horn and colon tissues [7]. Tissue type had a large effect on the extent of damage. Therefore, the authors stress that safe use of ABC requires an understanding of thermal effects in target tissues. In porcine small bowel and colon serosa, the TTD was compared for ABC and PJ devices [8]. The depth was more variable and power-dependent during the use of ABC (0.7–1.9 mm) compared to PJ (0.6–1.0 mm) devices. Predominant effects were coagulation and desiccation for the ABC and ablation for the PJ device.

APC (ARCO-MC, Söring) power setting and application time resulted in an increased TED in stomach, small intestines, and colon tissues [9]. Here, an effect of tissue type was not found. Changes in the probe angle (45°/90°) resulted in a change of the coagulation zone (oval/round) but did not alter the depth of thermal damage. In turn, APC (VIO 300D, ERBE) lesions were evaluated in rat peritoneum when rats were sacrificed 10 days after trauma induction [10,11,12,13]. Power setting affected the average lesion and inflammation depth, which ranged between 0.6–1.7 mm (10 W vs. 25 W, Student’s t-tests, *p* < 0.0001) [10]. In a pulsed APC setting (25 W), this was 1.3 mm [11]. The depth of tissue damage increased with pulse duration [14]. To reduce peritoneal adhesion formation, the peritoneum may be elevated with a water jet before applying APC (HybridAPC) [13]. The layer of water also acts as a heat sink, reducing the average lesion depth to 0.3 mm. Similarly, submucosal saline injections in porcine gastrointestinal tract tissues may be used to limit the depth of injury caused by APC [14,15,16].

The J-Plasma (20% power) TED was evaluated in vivo in porcine ovaries (0.11 ± 0.04 mm) and uterine horns (0.42 ± 0.13) [17]. The injury was smaller than that created with monopolar and bipolar ES devices. Furthermore, it was observed that the J-Plasma device (40% power, 4.0 l/min gas flow, 80 pulse setting) enabled the collection of peritoneal biopsies with cleaner edges compared to monopolar ES [18].

In short, tissue heating by ES devices decreases with depth along the current density, which is governed by device power. Plasma assistance may achieve a more uniform energy delivery to tissue. Thermal effects also depend on electrical resistivity and tissue geometry, introducing tissue type dependencies [7,17]. Finally, the extent of damage relies on exposure time [6]. In gynaecological tissues, the TED was found to range between 0.1–2.4 mm. This may be reduced or controlled with submucosal saline injections [13].

**Table 2:** Depth of tissue effects are reported in literature with either a range or mean ± std notation. Experimental models are annotated as: H = human, P = porcine, R = rodent, 1 = in-vivo, 2 = in-vitro/ex-vivo, O = ovary, U = uterus, F = fallopian tube, I = intestines, S = sigmoid colon, E = endometrium, Pe = peritoneum, M = intestinal mesentery and Om = omentum. Tissue effects are measured as total depth of damage (TDD), vaporization depth (VD), or thermal effect depth (TED).

Author, Year [Ref.]	Experimental Setting		Device Use Parameters					Thermal Tissue Effect Metrics			
	Tissue Models	Patients/ Samples	Type ^a^	Power	Time (s)	Dist. (mm)	Flow (L/min)	TDD (mm)	VD (mm)	TED (mm)	
										Eschar	Necrosis
Electrosurgical (plasma-assisted) devices											
Bristow, 2001 [6]	H2-O ^b^	1/16 1/16 1/16 1/16 1/16 1/16	ABC	60 W 60 W 80 W 80 W 100 W 100 W	1 5 1 5 1 5	<10 <10 <10 <10 <10 <10	7 7 7 7 8 8	1.7 ± 0.3 2.4 ± 0.4 2.2 ± 0.3 3.7 ± 0.7 3.2 ± 0.4 5.6 ± 0.5	0.6 ± 0.2 - - - - 3.2 ± 0.3	0.5 ± 0.1 - - - - 1.1 ± 0.2	0.6 ± 0.2 - - - - 1.3 ± 0.3
Gale, 1998 [7]	P1-U P1-I	2/15 2/24	ABC	60–80 W 40–80 W	1–5 1–5	5–10 5–10	4 4	- -	- -	- -	0.5–1.0 0.4–2.1
Tanner, 2017 [8]	P1-I	1/16	ABC	30–90 W	1	5–10	-	0.7–1.9	-	-	-
Johanns, 1997 [9]	H2-I	-/10	APC	40–155 W	1–10	5	2–7	-	-	0.1–2.4	
Kraemer, 2011 [10]	R1-Pe	9/36 9/36	APC	10 W 25 W	4 4	2–3 2–3	0.3 0.3	- -	- -	0.6 ± 0.4 ^e^ 1.7 ± 1.2 ^e^	
Kraemer, 2014 [11]	R1-Pe	16/62	APC	25 W	4	2–3	0.3	-	-	1.3 ± 1.1 ^e^	
Kraemer, 2014 [12]	R1-Pe	16/64	APC	25 W	4	2–3	0.4	-	-	2.2 ± 0.7 ^e^	
Kraemer, 2018 [13]	R1-Pe	24/48	Hybrid APC	25 W	-	3	0.4	-	-	0.3 ± 0.1 ^e^	
Llarena, 2019 [17]	P1-O P1-U	8/15 8/32	J-Plasma	20% 20%	5 5	5 5	- -	- -	- -	0.11 ± 0.04 0.42 ± 0.13	
Deb, 2012 [19]	H1-U H1-O H1-F	15/15 10/10 10/10	HTC	4 W 4 W 4 W	- - -	5 5 5	- - -	0.7 ± 0.2 ^f^ 0.7 ± 0.2 ^f^ 0.6 ± 0.1 ^f^			
Neutral argon plasma devices											
Tanner, 2017 [8]	P1-I	1/24	PJ	10–30%	1	5	-	0.6–1.0	-	-	-
Deb, 2012 [19]	H1-U H1-O H1-F	15/15 10/10 10/10	PJ	20% 20% 20%	5 5 5	5–10 5–10 5–10	- - -	0.6 ± 0.2 ^f^ 0.6 ± 0.1 ^f^ 0.6 ± 0.2 ^f^			
Madhuri, 2014 [20]	H1-U ^c^ H1-S ^b^	3/48 3/48	PJ	10–60% 10–80%	1–9 1–4	10 10	- -	- -	0.2–3.5 0.2–3.5	0.2–1.0 0.1–0.4	
Sonoda, 2010 [21]	H2-O H2-Pe ^b^	4/48 4/48	PJ	70–85% 70–85%	2 4	10 10	- -	- -	1.6–2.2 2.7–4.0	0.11–0.12 0.13–0.15	
Roman, 2011 [22]	H1-O ^d^	8/10	PJ	40%	1	5	-	-	-	-	0.1 ± 0.1
Nieuwenhuyzen-de Boer, 2022 [23]	H1-U/O/ I/Pe/Om/M	17/106	PJ	10%	3–4	5–10	-	-	-	0.15	

^a^ Device type abbreviations include: ABC (Argon Beam Coagulator), APC (Argon Plasma Coagulation), HTC (Helica Thermal Coagulator), and PJ (PlasmaJet). ^b^ Serous carcinoma, ^c^ Leiomyoma, ^d^ Endometrioma. ^e^ Evaluated 10 days after trauma induction. ^f^ Unclear histological definition of thermal damage.

#### 3.1.2. Neutral Argon Plasma Devices

The PJ relies on collisions of energetic particles with tissue. Transfer of kinetic energy into heat happens directly at the plasma–tissue interface. Thermal effects of the PJ were assessed in uterine leiomyomas and sigmoid bowel serous carcinoma samples [20]. Increasing the power and exposure time resulted in more tissue vaporization (0.2–3.5 mm), whereas the depth of the eschar remained relatively constant (<1 mm). In a similar approach, tumours were harvested during ovarian and peritoneal cancer surgery in four women [21]. Exposure time had a larger effect on vaporization depth than power. In addition, the PJ and HTC were compared in uterine, ovarian and fallopian tube tissues in 15 women undergoing hysterectomy [19]. The PJ and HTC resulted in a similar TDD between 0.6–0.7 mm. However, the width of the thermally affected zone did differ and was between 4.1–4.7 mm and 5.9–7.7 mm, respectively (Student’s *t*-test, uterus: *p* < 0.001, ovary: *p* < 0.001, fallopian tube: *p* = 0.034). The PJ device caused coagulation, desiccation and vaporization, whereas the HTC device caused coagulation and haemostasis. In a series of 10 samples collected from 8 women with ovarian endometriomas, a mean depth of necrosis of 145 µm was found [22]. Finally, thermal effects of PJ and electrosurgical devices were compared in a series of 106 tissue samples of 17 ovarian cancer patients undergoing cytoreductive surgery [23]. Devices were used in vivo on to-be-resected tissues of reproductive organs, and intestines as well as on membranes. The mean TED of the PJ (0.15 mm, range 0.03–0.60 mm) was lower than that of the electrosurgical unit (0.33 mm, range 0.08–1.80 mm) (Mann–Whitney U test, *p* < 0.001). 

In short, the PJ TED remains below 1.0 mm, and VD depends on application time, and to a lesser extent power [21]. For divergent plasma beams, particle density is higher close to the device tip. Energy delivered to the tissue surface may therefore also depend on distance to tissue. Tissue dependencies may be caused by variations in biomechanical tissue properties. However, so far, there are no indications that these effects can become clinically relevant [19].

#### 3.1.3. Cold Atmospheric Plasma Devices

CAP (kINPen) devices aim to trigger biological redox reactions that cause cancer-specific apoptosis or inhibit spread of disease. Furthermore, PAM is under evaluation to reduce post-operative peritoneal adhesions [24]. It appears that oxygen species are less capable of penetrating tissue than nitrogen species [25]. Some species can penetrate 1.25 mm of biological tissue. However, in absence of thermal injury, quantifying the effects of remaining species on tissues is complex. In a pancreatic cancer model (Colo-357 chorio-allantoic membrane assay), the reported effect of kINPen APPJ exposure (20 s) was cell death induced in the upper 3–5 cell layers, reaching approx. 50 µm [26]. By means of Raman imaging, differences in DNA and lipids in the superficial and basal cell layers were identified over an average thickness of 270 µm, in a series of 10 cervix uteri tissue samples treated with the kINPen [27]. Raman data suggest that reactive species can cross the full thickness of human mucosa [28]. However, the completeness of treatment in these layers remains unclear. As a concept, it is often suggested that PAM can facilitate dose delivery via needle injection [29]. Although the direct reach of plasma particles may be limited, tissue effects may be more extensive [30]. It is theorized that cell-to-cell communication plays a role in the spread of plasma effects [30,31].

In short, very few data on affected depths or volumes of tissue are available for CAP devices. In absence of thermal effects, it is complex to (histologically) identify and assess these zones. Current estimates rest on computer models and gelatine or agarose gel models, while in vivo mechanics and CAP effectiveness need to be better understood [32,33].

### 3.2. Device Function: Type of Tissue Effects

#### 3.2.1. Cutting, Incision and Dissection

The PlasmaBlade is an electrosurgical soft tissue dissection tool with a plasma rim generated around the blade. It operates at a lower temperature than traditional ES devices and causes less thermal damage [34]. The device is mostly investigated in plastic and reconstructive surgery. It was used to create incisions for caesarean section in a randomized study in 40 women [35]. Cosmetic outcomes were compared to those of traditional scalpels and rated after six months by patients and dermatologists using Patient and Observer Scar Assessment Scale scores. The PlasmaBlade resulted in a more favourable score (30.9 ± 11.9) than the scalpel (48.9 ± 18.6). The Canadian Agency for Drugs and Technologies in Health filed a review of literature on the PlasmaBlade in 2019, identifying the need for more rigorous comparative research and determining its cost-effectiveness [36].

The ABC was used for laparoscopic dissection of pelvic (*n* = 61), pelvic and aortic (*n* = 35), and aortic (*n* = 18) lymph nodes [37]. Rates for complications and conversions to laparotomy were 7% and 8%, respectively. Furthermore, the PJ device has been used for groin node dissection (*n* = 3), wide local excision of vulva (*n* = 1), ovarian cyst removal (*n* = 22), myomectomy (*n* = 2), and tubal surgery—salpingectomy or salpingotomy (*n* = 6) [38]. However, detailed clinical processes or outcomes were not reported. 

In short, comparative data of plasma-based devices used for cutting of gynaecological tissues are currently insufficient to assess their effectiveness relative to that of conventional devices.

#### 3.2.2. Coagulation

Gynaecological cancer management can include radiotherapeutic components. In this context, the American Society of Colon and Rectal Surgeons advises endoscopic APC for the treatment of rectal bleeding induced by chronic radiation proctitis, as it leads to a meaningful decrease in bleeding in 79–100% of patients [39]. They advise power settings of 40–60 W, gas flow rates of 1–2 L/min, and applications in 1–2 s pulses. The treatment is considered safe, with 19–35% of patients experiencing self-limiting early complications (e.g., proctalgia, rectal mucous discharge, incontinence, gas emphysema) and 0–3% of patients experience late complications (rectal stenosis) [40,41,42]. A pooled analysis of 33 studies (957 patients) showed an overall clinical success rate of 87% and a serious adverse event (colonic fistula, perforation, explosion, or stricture) rate of 4% [43]. Abdominal, rectal, and anal pain were the most common APC-related adverse events. To prevent excessive bowel distention, alleviate pain, and reduce the risk of post-procedural discomfort, it is advised to periodically remove the argon gas flow.

The European Society of Gastrointestinal Endoscopy considers APC the treatment of choice for angioectasia in the gastrointestinal tract [44]. The perforation incidence after APC (Söring) treatment of the colon was 0.3% (1/373 patients) [45]. After APC (ERBE) treatment of 100 patients with angiodysplasia, the probability of remaining free of rebleeding was high: 98% after 1 year and 90% after 2 years [46]. In a group of 94 patients with angiodysplasia, 234 visible lesions were coagulated successfully with APC (ERBE, PRECISE mode) [47]. After the procedure, no perforation, active bleeding, or tissue carbonization was reported. At 6 months follow-up, rebleeding was seen in 19% of patients, and new lesions in the same area were seen in 16% of patients.

In short, coagulation with plasma-based devices largely rests on APC, and the device is used mostly on tissues of the colon and rectum. Other plasma-based devices may induce coagulation, but their clinical effectiveness is less well studied. APC is the modality of choice in clinical guidelines for the management of rectal bleeding caused by radiation proctitis [39] as well as in the treatment of angioectasia [44]. Clinical outcomes were studied with decent patient cohorts and follow-up times. Comparative studies with other devices that may induce coagulation remain limited.

#### 3.2.3. Ablation I: Endometriosis

The National Institute for Health and Care Excellence reviewed the use of laparoscopic helium plasma coagulation for the treatment of endometriosis in 2006. At the time, no major safety concerns were found, yet evidence of the procedural efficacy was still insufficient. The same year, the results of a series of 1060 women treated laparoscopically with HTC were reported [48]. The complication rate was low at 0.1%, and satisfactory pain relief was seen in 70% of patients [49,50]. In a recent randomized controlled trial, HTC and traditional ES devices were used in 192 women with mild-to-moderate endometriosis [51]. Cyclical pain and dyspareunia were assessed with visual analogue scales (VAS) at 6, 12 and 36 weeks after surgery. VAS scores were significantly lower (better) for electrosurgical than for HTC treatments. However, it was pointed out that treatment groups differed, i.e., the electrosurgical group involved excisions in 99% of cases compared to 60% in the HTC group [52].

The PJ is also being studied for endometriosis treatment. The depth of thermal effects is comparable to that of HTC [19], and is typically < 1 mm [53]. In a group of 34 patients, PJ treatment was considered safe and did not result in postoperative complications, problems with healing of the wound, long-lasting postoperative pain, or disease recurrence [54]. Using a 3D ultrasound-based assessment of ovarian volumes and antral follicle counts in 30 women, the PJ was compared to cystectomy. With counts of 5.5 ± 3.9 and 2.9 ± 2.4, the PJ was considered a tissue-sparing alternative [55]. In a follow-up study in 104 women treated with the PJ or cystectomy, no differences were found in pregnancy probability at 36 months (84.4% and 78.3%) after surgery [56]. It should be noted that both values are high. Finally, quality of life was compared using the Endometriosis Health Profile 5 questionnaire in 52 women with deep endometriosis treated with shaving, shaving with PJ use, and resections [57]. Both the PJ and resection methods resulted in better quality of life outcomes compared to shaving. 

In short, there is an ongoing debate on the need for ablation or deep excision of endometriosis to achieve pain and dyspareunia management. This debate extends to the optimal devices for treatment. A competing factor for deep excisions is the preservation of ovarian parenchyma in women with a wish to conceive.

#### 3.2.4. Ablation II: Carcinomatosis

One of the first gynaecological applications of plasma-based devices was cytoreductive surgery or debulking of advanced ovarian cancer [1]. In this study, seven patients were treated with ABC, of which four were left with no visible remaining disease. At a mean follow-up time of 33 months, 5 patients survived of which 4 disease-free. In a group of 45 patients, ABC improved the rate of achieving complete cytoreduction to 74% [58]. Furthermore, using ABC and the ES loop excision procedure, laparoscopic debulking was performed in 36 patients with stage III or IV ovarian cancer. The approach was considered feasible (two conversions to laparotomy, 6%), successful (complete cytoreductive surgery, 94%) and safe (two complications, 6%: 1 epigastric hematoma, 1 cystotomy) [59]. 

As an alternative, the PJ has been used to treat advanced-stage ovarian cancer in groups of 19 patients [60], 51 patients [61], and 87 patients [62]. Carcinomatosis was removed from the peritoneum, bowel serosa, intestinal mesentery, diaphragm, and liver surface. In these studies, complete cytoreductive surgery was achieved in 100%, 78%, and 99% of patients, respectively. Surgical complications were limited. Most often reported was the need for blood transfusions. In a multicentre randomized controlled clinical trial with 327 patients (intervention group with PJ: 157, control group without PJ: 170), per-protocol complete cytoreductive surgery was achieved in 85.6% and 71.5% of patients (chi-squared test, *p* = 0.005), respectively [2]. There were no significant differences in blood perfusion, hospital time, number of colostomies (9 vs. 20, *p* = 0.169), or surgical complication rates. In case of peritoneal carcinomatosis (≥50 lesions on peritoneum, diaphragm, or mesentery), complete cytoreductive surgery was achieved in 72.2% and 51.5% in intervention and control groups, respectively. Six months after surgery, patients in the intervention group reported a better perceived health score compared to the control group (EQ-VAS: 73.4 vs. 69.0, *p* = 0.029, mean EQ-5D-5L health state: 0.80 vs. 0.76, *p* = 0.049). 

In short, early studies with the ABC and recent studies with the PJ have addressed plasma use for cytoreductive surgery for ovarian cancer patients. A recent randomized controlled clinical trial showed that PJ treatment may increase surgical completeness. Currently, there is still a need for long-term follow-up to assess overall and disease-free survival after these treatments.

#### 3.2.5. Topical Therapy I: Gynaecological Intraepithelial Neoplasia

The ABC was evaluated for the local treatment of vulvar intraepithelial neoplasia (VIN) in 29 patients [63]. Human papillomavirus status was known for 10 patients (9 positive, 1 negative). After a mean follow-up of 35 months, 52% of patients had no recurrence, with a mean time to recurrence in the other 48% of 23 months. ABC was considered an alternative treatment that may improve cosmesis and organ and form conservation. Alternatively, the PJ was used in a series of eight patients with recurrent and multifocal uVIN or perianal intraepithelial neoplasia [64]. Of this group, six patients required repeat PJ treatment. At a median follow-up of 269 days, excellent macroscopic and symptomatic improvements were reported. Furthermore, there are indications that cervical intraepithelial neoplasia (CIN) can be treated with CAP. In a study in 48 premenopausal women with histologically confirmed CIN I (37%) and II (63%), CAP induced a remission of CIN after 3 months in 81% of patients, with 93% sustainability after 6 months [65,66]. The treatment also resulted in a normalization of high-risk HPV (71% to 11%) and PAP smear testing. Finally, CAP has also been tested for the treatment of chronic vulvar pruritus in 3 patients [67]. After three 90 s sessions per week, for six weeks, a significant reduction of itch was reported, whereas clinical signs improved only slightly. 

In short, plasma-based treatment of vulvar, anal and cervical intraepithelial neoplasia has been tested with a view to preservation of tissue form and function. So far, different plasma-based devices (ABC, PJ, and CAP) were used. However, the application field is new, and there is a need for randomized controlled trials and elaborate comparative studies to address cost-effectiveness as well as risks of disease recurrence.

#### 3.2.6. Topical Therapy II: Selective Cancer Treatment

Although our literature search is focused on clinical studies, a short summary of in-vitro CAP developments is in order due to the fast developments and important prospects of this field. CAP uses the difference in sensitivity to reactive oxygen and nitrogen species to reduce viability or induce apoptosis of specific cell types. Typically, researchers are in search of a sweet spot in plasma conditions at which cancer cells die and nearby healthy cells survive. Detailed biochemical pathways through which CAP (potentially) work have been previously reviewed [29,68]. In vitro studies have been conducted on a wide range of gynaecological cell lines (see also Table 1 in the work of Zubor et al. [68]) of cervical cancer [69,70,71,72], ovarian cancer [73,74,75,76,77,78], and colon cancer [79,80,81], see Table 3. With an aim to increase differences in cell response between healthy and cancer cells, more intricate applications are in development, combining CAP with hyperthermia [82], radiotherapy [72], or photodynamic therapy using targeted polymeric nanoparticles [83]. 

In addition to curative potential, anti-proliferative effects of CAP on cancer cells have been studied—for instance, on cervical cancer cell lines using the MABS device [84]. With a CAP DBD device, suppressed migration and invasion of cervical cancer HeLa cells was shown [85]. Antiproliferative effects of CAP were also demonstrated in ovarian cancer cell lines [86]. In addition, PAM inhibited dissemination of ovarian cancer (ES2) cells in an in-vivo mouse model of intraperitoneal metastasis, resulting in prolonged survival [87,88].

In short, CAP may enable selective killing or antiproliferation of cancer cells. Definite proof of treatment selectiveness remains scarce, as culture media used for cancerous and non-cancerous cell lines often differ, which can affect the stability of reactive oxygen and nitrogen species [89]. Additionally, it is uncertain whether changes in protein expression realised in cell cultures can be transferred to in vivo treatment [90]. Finally, the CAP-affected zone needs to be better understood, and methods for local CAP delivery may need to be developed. While preclinical cell line studies in the past two decades were abundant, there is a pressing need for translational and clinical studies. These will determine the future role of CAP as a first line or adjuvant treatment for dysplastic tissues.

## 4. Discussion

Plasma-based devices are increasingly used in gynaecological treatments. The aim of this review was to delineate tissue effects of plasma devices as a function of device settings and parameters of use. This was done for various tissues of the female pelvis, include the uterus, ovaries, cervix, vagina, vulva, colon, omentum, mesenterium, and peritoneum. 

Three device classes were identified in this review: electrosurgical (ES, plasma-assisted) devices aim to achieve electrothermal tissue heating; neutral argon plasma (NAP) devices aim to achieve tissue heating by transferring kinetic particle energy; and cold atmospheric plasma (CAP) devices aim to trigger biochemical cell responses. All three classes focus on targeted therapies, albeit creating different tissue effects and being developed for different fields of application. 

A critical determinant of plasma applicability is the depth or volume of tissue effects required to achieve complete treatment and limit damage to healthy tissues, e.g., to prevent perforations or limit toxicity. Thermal effects were reported as total depth of damage (TDD), vaporization depth (VD), or thermal effect depth (TED), which includes layers of eschar and necrosis. In practice, VD can be macroscopically inspected, whereas TED is the subsurface damage that is not directly visible. Overall, we found that tissue effects of ES devices depend on device power, exposure time, and tissue type and shape. Plasma-assistance may improve the uniformity of ES energy distributions. The TED of plasma-assisted ES devices in pelvic tissues was found to range between 0.1–2.4 mm. In turn, NAP and CAP devices rely on direct plasma–tissue interactions, and their effects remain more superficial. The TED of NAP devices was <1.0 mm, while the VD increased with application time and to a lesser extent with power settings. Here, tissue dependencies appeared to be limited. Finally, CAP effects are non-thermal. So far, few studies have provided quantitative assessments of affected treatment zones. 

When reporting the extent of thermal effects, we advocate adding visual definitions in exemplar histological images. Unambiguous definitions are needed to enable inter-study comparison of results. In particular, it was not always clear whether vaporization effects were included in thermal damage measures. In addition, thermal effects may be reported by describing the (proportion of) tissue layers affected [16,91,92]. This qualitative approach is pragmatic and valuable to construct guidelines for device use in a specific clinical context. However, it prevents a broader and quantitative comparison of device functioning, as tissue layer types and thicknesses differ per organ.

Plasma-based device functions in gynaecology have included coagulation, ablation, and—to a lesser extent—the cutting of tissues. In addition, topical use of plasmas has been explored for non-surgical or adjuvant clinical therapies. Plasma-assisted ES was used mostly for coagulation in colorectal procedures to treat bleeding after radiation proctitis and angioectasia. In these fields, the APC has been an established device advocated in multiple clinical guidelines. In turn, NAP is predominantly investigated for the treatment of endometriosis or carcinomatosis. Evidence of device effectiveness is gradually accumulating, yet there is a need for additional randomized clinical trials, comparison studies, and longitudinal patient follow-up and quality of life studies. Finally, CAP is currently in development for the selective killing of cancer cells. However, its physicochemical and immunomodulatory effects need to be better understood. So far, it remains uncertain whether changes in protein expression in cell cultures can be transferred to in vivo treatment [90]. This stresses the need for clinical and translational studies to assess true effectiveness and selectiveness of CAP treatments in patients.

This systematic literature review has several limitations. We have presented data on the (non-)thermal effects of plasma devices on organs that may be treated by a gynaecologist. However, we did not discriminate between the ways in which these organs were approached. For example, the colon may be approached facing the serosa during cytoreductive surgery or facing the mucosa during endoscopic procedures. Consequently, application fields may have extended beyond those typically performed by a gynaecologist. However, in absence of studies comparing thermal effects after treatment of mucosal and serosal organ sides, we have decided to not let this restrict the search results.

In some hospitals, breast cancer treatment falls in the domain of the gynaecologist. In this field, (non-)thermal plasma devices have also been evaluated. Based on a study with 80 patients undergoing breast-conserving surgery, it was suggested that the ABC may reduce the development of haematomas or seromas [93]. Furthermore, the PlasmaBlade was compared to conventional diathermy in a study with 108 abdominal-based free-flap breast reconstruction patients [94]. The PlasmaBlade resulted in less abdominal seromas at 2-week follow-up. In recovery (day 0), the PlasmaBlade did result in a higher median pain score (4, IQR 1.0–6.0) compared to diathermy (2, IQR 1.0–5.0). Finally, CAP applications for breast cancer therapy were recently reviewed by Chupradit et al. [95].

An important factor that is unaddressed in this review is device costs and cost-effectiveness. Studies on these topics remain scarce [36]. Cost calculations should include procurement (including infrastructural changes) and running costs (including maintenance and consumables) [96], and may involve costs of both inpatient and outpatient care [97]. Most plasma-based devices appear to be made for single-time use. Differences in costs need to be weighed against gains in treatment outcomes, (post-)operative risks, and differences in the patient’s quality of life.

## 5. Conclusions

Plasmas have been used in various forms and with various functions in gynaecology. Plasma devices can be divided into three classes: making use of electrosurgical energy (plasma-assisted), kinetic energy of neutral argon plasma particles, or biochemical reactions of cold atmospheric plasmas. In this review, historical and potentially future plasma applications are summarized, and the depth of effects is evaluated in tissues of the uterus, ovaries, cervix, vagina, vulva, colon, omentum, mesenterium, and peritoneum. The depth of treatment of electrosurgical devices relies on electric current dispersion in tissue. In turn, direct plasma–tissue interactions tend to remain superficial. In terms of device applications, argon plasma coagulation is used mainly in colorectal procedures, including the treatment of bleeding after radiation proctitis and the treatment of angioectasia. Neutral argon plasma is predominantly evaluated for the treatment of endometriosis and carcinomatosis. Finally, cold plasmas are evaluated for the treatment of intraepithelial neoplasia, and for selective treatment of cancer cells. This review identifies upcoming and potentially high-gain applications of plasma in the field of gynaecology, of which the therapeutic effectiveness needs to be examined in translational and clinical studies.

## Figures and Tables

**Table 1:** An overview of clinically (gyn.) tested plasma-based devices, grouped by plasma type.

Type	Form	Brand
Electrosurgery (plasma-assisted)	Beam	ABC, ConMed, Largo, FL, USA. APC, ARCO series, Söring GmbH, Quickborn, DE. APC, ERBE GmbH, Tübingen, DE. HTC, Helica Instruments Ltd., Currie, UK. HybridAPC, ERBE GmbH, Tübingen, DE. J-Plasma, Apyx Medical, Clearwater, FL, USA. MABS, KLS Martin Group, Tuttlingen, DE.
	Rim	PlasmaBlade (PEAK), Medtronic, Dublin, IE.
NAP Neutral argon plasma	Beam	PlasmaJet, Plasma Surgical, Atlanta, GA, USA.
CAP ^1^ Cold atmospheric plasma	Beam (APPJ)	kINPen, Neoplas med GmbH, Greifswald, DE.
	Surface (DBD)	PlasmaDerm, CINOGY System GmbH, Duderstadt, DE.
	Medium (PAM/PAL)	All non-commercial or not clinically (gyn.) tested

^1^ Or LTP (low-temperature plasma), NEAPP (non-equilibrium atmospheric pressure plasma).

**Table 3:** Overview of CAP devices and experimental models, annotated as: C = cancer cell line, R = rodent, 1 = in vivo, 2 = in vitro, O = ovary, Ce = cervix, Co = colon.

Author, Year [Ref.]	Experimental Setting		Device Type
	Tissue Models	Pelvic Organ (Cancer) Cell Lines	
Reduced viability/apoptosis			
Ahn, 2011 [69] Li, 2017 [70] Wenzel, 2020 [28] Jezeh, 2020 [71] Kenari, 2021 [72]	C2-Ce	HeLa HeLa, HFB SiHa HeLa, HFB HeLa	Custom APPJ (N_2_/air) Custom DBD (N_2_) and PAL (NO) Custom APPJ VIO3 APC ERBE (Ar) Custom APPJ (He) and PAM (He/He + O_2_) Custom APPJ and DBD (Ar + air)
Iseki, 2012 [73] Utsumi, 2013 [74] Utsumi, 2014 [75] Bekeschus, 2018 [76] Bisag, 2020 [77] Rasouli, 2021 [78]	C2-O	SKOV-3, HRA, WI-38, MRC-5 NOS2, NOS3 TOV21G, SKOV-3, ES-2, NOS2, OHFC, HPMC SKOV-3, OVCAR-3 SKOV-3, OV-90, HOSE, F1, F2 SKOV-3, A2780 CP, GC	Custom APPJ (Ar) Custom PAM (Ar) Custom PAM (Ar) kINPen MED, APPJ (Ar) Custom/AlmaPlasma PAL (air) Custom APPJ (He) and PAM (He)
Tuhvatulin, 2012 [79] Kumara, 2016 [80] Choi, 2017 [81]	C2-Co	HCT116 SNUC5 HCT116	MicroPlaSter β, APPJ (Ar) Custom DBD (O_2_ + Ar) Custom DBD (N_2_)
Reduced proliferation/growth			
Feil, 2020 [84] Li, 2016 [85]	C2-Ce	SiHa, CaSki, C-33-A, DoTc2 4510, NCCT HeLa	MABS, APPJ (Ar) Custom DBD (He)
Koensgen, 2017 [86] Nakamura, 2017 [87] Nakamura, 2021 [88]	C2-O	SKOV-3, OVCAR-3, TOV-21G, TOV-112D ES2, SKOV3, HPMC ES2, SKOV3, OV90, OVCAR3, CAOV3	kINPen MED, APPJ (Ar) Custom PAM (Ar) Custom/Tough Plasma PAM/PAL (Ar)
Utsumi, 2013 [74] Nakamura, 2017 [87] Nakamura, 2021 [88]	R1-O	NOS2 ES2 ES2	Custom PAM (Ar) Custom PAM (Ar) Custom PAM (Ar)

## Abbreviations

ABC	Argon Beam Coagulation
APC	Argon Plasma Coagulation
APPJ	Atmospheric Pressure Plasma Jet
CAP	Cold Atmospheric Plasma
CIN	Cervical Intraepithelial Neoplasia
DBD	Dielectric Barrier Discharge
ES	Electrosurgery
HTC	Helica Thermal Coagulator
NAP	Neutral Argon Plasma
PAM/PAL	Plasma Activated Medium/Plasma Activated Liquid
PJ	PlasmaJet
TDD	Total Depth of Damage
TED	Thermal Effects Depth
VD	Vaporization Depth
VIN	Vulvar Intraepithelial Neoplasia
